# ImmunoPET helps predicting the efficacy of antibody-drug conjugates targeting TENB2 and STEAP1

**DOI:** 10.18632/oncotarget.8390

**Published:** 2016-03-26

**Authors:** Simon-Peter Williams, Annie Ogasawara, Jeff N. Tinianow, Judith E. Flores, David Kan, Jeffrey Lau, MaryAnn Go, Alexander N. Vanderbilt, Herman S. Gill, Li Miao, Joshua Goldsmith, Bonnee Rubinfeld, Weiguang Mao, Ron Firestein, Shang-Fan Yu, Jan Marik, Anton G.T. Terwisscha van Scheltinga

**Affiliations:** ^1^ Genentech Research and Early Development, Genentech, Inc., South San Francisco, CA, 94080, USA; ^2^ Department of Clinical Pharmacy and Pharmacology, University Medical Center Groningen, University of Groningen, Groningen, 9700RB, The Netherlands

**Keywords:** antibody-drug conjugates, immunoPET, TENB2, STEAP1, zirconium-89

## Abstract

The efficacy of antibody-drug conjugates (ADCs) targeted to solid tumors depends on biological processes that are hard to monitor *in vivo*. ^89^Zr-immunoPET of the ADC antibodies could help understand the performance of ADCs in the clinic by confirming the necessary penetration, binding, and internalization. This work studied monomethyl auristatin E (MMAE) ADCs against two targets in metastatic castration-resistant prostate cancer, TENB2 and STEAP1, in four patient-derived tumor models (LuCaP35V, LuCaP70, LuCaP77, LuCaP96.1). Three aspects of ADC biology were measured and compared: efficacy was measured in tumor growth inhibition studies; target expression was measured by immunohistochemistry and flow cytometry; and tumor antibody uptake was measured with ^111^In-mAbs and gamma counting or with ^89^Zr-immunoPET. Within each model, the mAb with the highest tumor uptake showed the greatest potency as an ADC. Sensitivity between models varied, with the LuCaP77 model showing weak efficacy despite high target expression and high antibody uptake. *Ex vivo* analysis confirmed the *in vivo* results, showing a correlation between expression, uptake and ADC efficacy. We conclude that ^89^Zr-immunoPET data can demonstrate which ADC candidates achieve the penetration, binding, and internalization necessary for efficacy in tumors sensitive to the toxic payload.

## INTRODUCTION

Antibody-drug conjugates (ADCs) are a promising new class of anti-cancer therapeutics that aim to selectively deliver potent toxins to tumor cells by using monoclonal antibodies (mAbs) as targeting moieties. This should provide an improved therapeutic window in patient tissues expressing the cognate antigen. Multiple ADCs are in development for a variety of solid and hematological cancer indications [1]. The approach has already shown significant promise: recently-approved ADC molecules include brentuximab vedotin (anti-CD30-MMAE) for Hodgkins lymphoma and trastuzumab-emtansine (T-DM1) for metastatic breast cancer [2-4].

ADC target antigens are typically chosen because they exhibit tumor-specific expression and, if internalized, represent a selective entry portal into cancer cells [1]. However they are not necessarily molecular drivers of cancer, and consequently there may be no selection pressure resisting the loss of target expression in a new metastatic or post-treatment environment. Such a loss of expression could impair the ADC efficacy and this potential adds to the importance of comprehensively assessing and re-assessing the tumor target status.

Positron emission tomography (PET) imaging is widely used for characterizing tumors, offering excellent sensitivity and relatively good resolution [5-10]. ImmunoPET is used for tracking and quantification of mAbs with PET *in vivo*. The availability of zirconium-89 (^89^Zr), and the adoption of standardized labeling protocols are enabling clinical mAb imaging with immunoPET to become more widespread, offering the potential for whole-body non-invasive quantification of mAb uptake in normal and tumor tissues [5-10]. Because of the mechanistic importance of mAb delivery and tumor uptake to ADC therapies, immunoPET may be of particular value because the PET isotope is delivered to the cell by the same mAb and internalization mechanisms as the toxin. Thus an imaging signal in the tumor reveals processes necessary (but not sufficient) for drug delivery – a combination of tissue exposure, antigen binding, and antibody internalization [11].

For prostate cancer, TENB2 and STEAP1 have been recently identified as targets of interest for ADC development [12-14]. TENB2, a trans-membrane protein containing an epidermal growth factor-like motif and two follistatin-like domains, is found to be over-expressed in clinical prostate carcinoma with particular prominence in high-grade tumors [12]. STEAP1, six-transmembrane epithelial antigen of the prostate-1, is an antigen that is over expressed in the majority of human epithelial prostate cancers [13]. Treatment for advanced prostate cancer is particularly challenging due to the large number and potential heterogeneity of metastatic growths in soft tissue and especially in bone [15].

Molecular imaging of antibody delivery might predict treatment effects and therefore support drug development of the ADCs targeting TENB2 and STEAP1. To test this we examined the tumor growth inhibition properties of ADCs targeting these antigens in a panel of patient-derived prostate cancer models. Results were compared with parallel immunoPET studies performed to quantify tumor uptake of the corresponding mAbs targeting TENB2 and STEAP1 in the same tumor models. Both efficacy and imaging studies used anti-gD as an isotype-matched non-specific control antibody.

## RESULTS

### Radiolabeling of TENB2, STEAP1 and gD targeting mAbs

^111^In and ^89^Zr radiolabeled mAbs targeting TENB2, STEAP1 and gD all showed greater than 90% radiochemical purity. Binding was essentially unaltered in the potency assays for anti-TENB2 (IC50 of 1.03 ± 0.17 versus 0.96 ± 0.09 nM before and after radiolabeling respectively) and anti-STEAP1 (2.50 ± 0.09 versus 1.95 ± 0.15 nM).

### Dose dependence of tumor uptake with ^111^In-labeled anti-STEAP1

**Figure 1:**
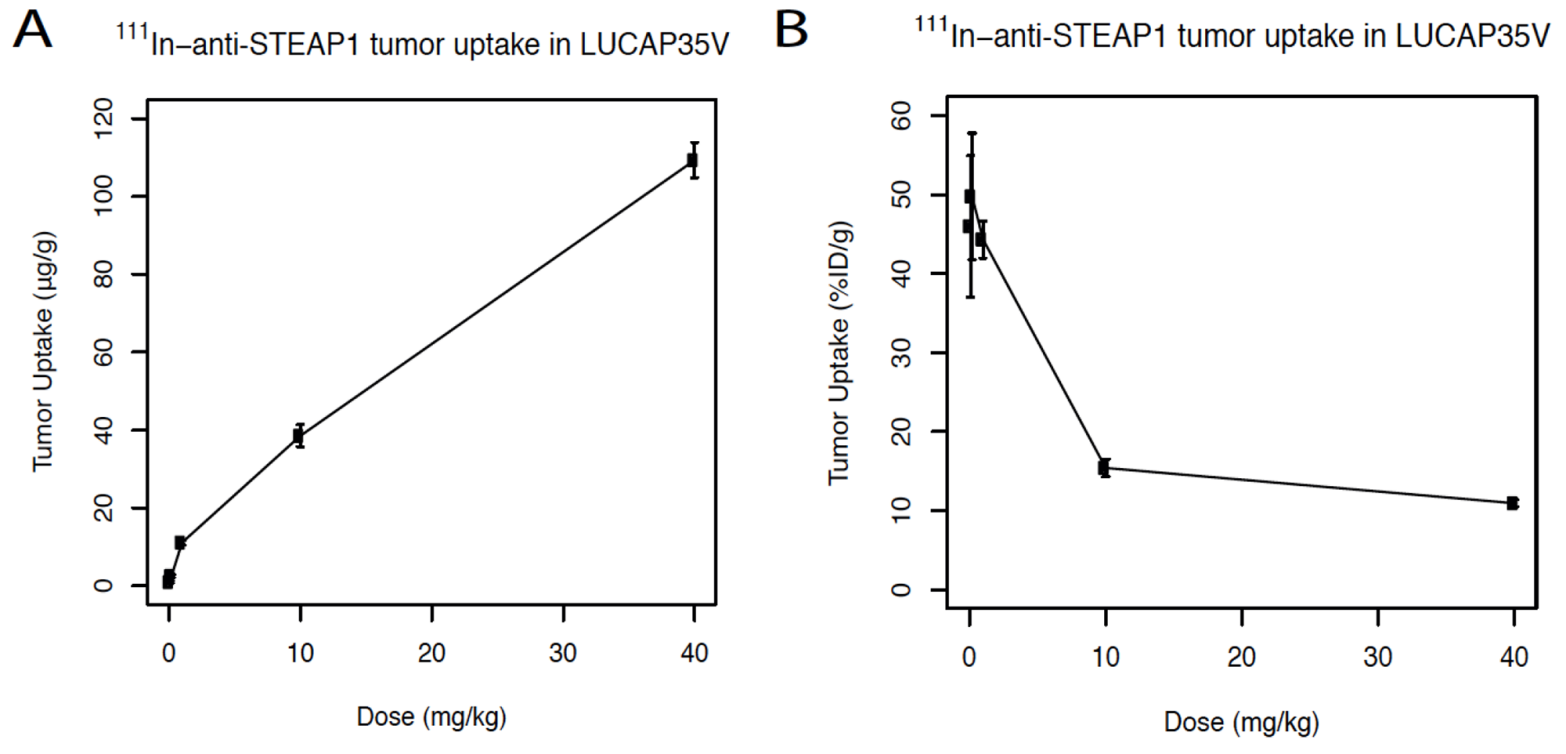
**A.** Dose dependent uptake studies of ^111^In-labeled anti-STEAP1. Absolute tumor uptake of ^111^In-anti-STEAP1 in LuCaP35V patient-derived xenografts expressed in μg/g. Mice received final dosages of 0.08 mg/kg, 0.2 mg/kg, 1 mg/kg, 10 mg/kg, or 40 mg/kg of anti-STEAP1. **B.** Fractional tumor uptake of anti-STEAP1 expressed as % of the injected dose per gram (%ID/g).

Dose-dependent tumor uptake was measured for ^111^In-anti-STEAP1. Figure 1A shows the absolute tumor uptake (μg mAb per g tumor tissue) which was highest for the highest dose level tested, 40 mg/kg. Figure 1B shows the relative tumor uptake (expressed as %ID/g) which was highest at the 0.08 mg/kg, 0.2 mg/kg and 1 mg/kg dose levels.

### Tracer tumor uptake and ADC tumor growth inhibition studies

**Figure 2:**
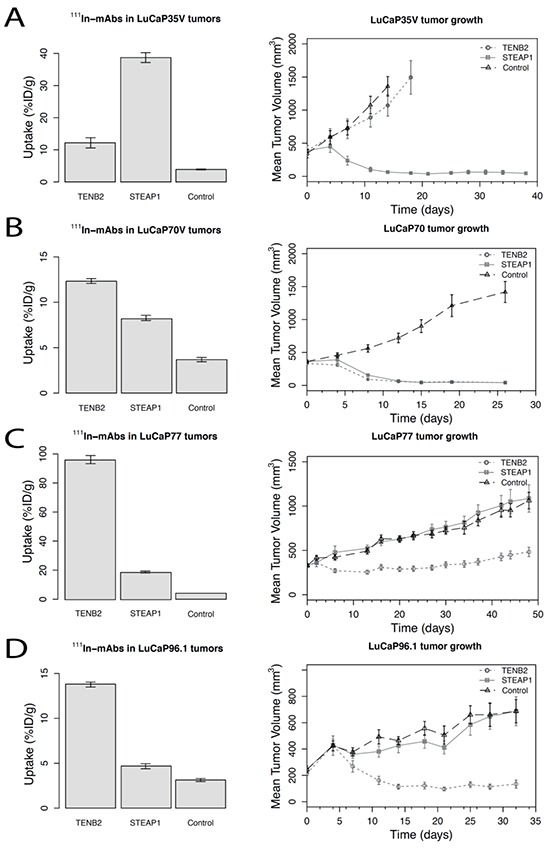
Tumor-tissue uptake of ^111^In-labeled STEAP1, TENB2, and control (gD) mAbs at 72 hours post-injection (5 mg/kg) (left column graphs) and tumor growth curves following dosing with the corresponding antibody-drug conjugates (5 mg/kg dosed once, on Day 1) (right column graphs) for four LuCaP tumor types **A.** LuCaP35V, **B.** LuCaP70, **C.** LuCaP77, and **D.** LuCaP 96.1 top to bottom. Data are shown as means with their corresponding standard errors of the mean. Numbers of animals per group were 4-5 for the ^111^In-mAb study and 8-10 for the tumor growth study.

Tumor tracer uptake of ^111^In labeled TENB2, STEAP1 and gD mAbs showed varying results (Figure 2). For all tumors, specific tumor uptake was observed relative to the non-specific gD control (P < 0.05). For TENB2, highest tumor uptake of 96.1 ± 2.8 %ID/g was measured in LuCaP77 tumors, the lowest (12.2 ± 1.6 %ID/g) in LuCaP35V. The anti-TENB2 tracer uptake in LuCaP70 was 12.3 ± 0.3 %ID/g and in LuCaP96.1 tumors 13.8 ± 0.3 %ID/g. For STEAP1 ADC tumor uptake, highest values (38.7 ± 1.5 %ID/g) were measured in LuCaP35V tumors, lowest tumor uptake in LuCaP96.1 tumors (4.7 ± 0.3 %ID/g). LuCaP70 and LuCaP77 tumors showed an uptake of 8.2 ± 0.3 %ID/g and 18.8 ± 0.6 %ID/g respectively.

Efficacy studies used tumor volumes measured over 4-7 weeks following TENB2 or STEAP1 ADC MMAE-conjugated drug treatment. LuCaP70 and LuCaP96.1 tumors were sensitive for TENB2 ADC treatment, whereas in LuCaP77 efficacy of this ADC was relatively poor as tumors started to regrow. STEAP1 ADC was efficacious in LuCaP35V and LuCaP70 tumors. In each LuCaP tumor line, the highest tumor uptake of mAb corresponded with the greatest on-treatment reduction in tumor volume.

### PET imaging

**Figure 3:**
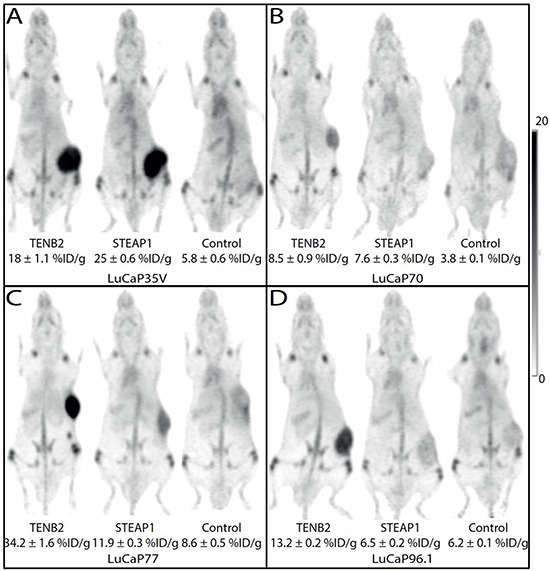
Maximum intensity projections from mouse coronal views of ^89^Zr-immunoPET images obtained 5 days post injection of TENB2, STEAP1 and control (gD) mAbs in four LuCaP tumor types **A.** LuCaP35V, **B.** LuCaP70, **C.** LuCaP77, and **D.** LuCaP96.1. Tumors are growing on the flank visible as areas of intense dark uptake in some images. The intensity scale bar is calibrated as percentage of the injected dose per gram (%ID/g).

Figure 3 shows representative images (maximum intensity projections), obtained five days after tracer injection and selected for tumor uptake closest to the median values in each group. Tumor uptake was higher in mice administered ^89^Zr–anti-TENB2 or ^89^Zr–anti-STEAP1 compared to mice given the control mAb ^89^Zr–anti-gD (p < 0.05); the exception was ^89^Zr-anti-STEAP1 uptake in LuCaP96.1 tumors which was no higher than control. ^89^Zr tumor uptake as determined by PET quantification correlated with the ^111^In labeled mAb biodistribution studies (R^2^ = 0.85).

### *Ex vivo* tumor analysis

**Figure 4:**
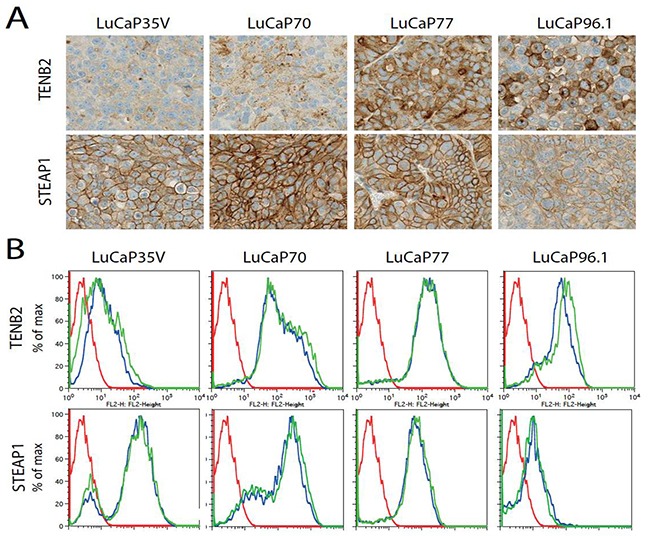
*Ex vivo* analysis of TENB2 and STEAP1 tumor expression **A.** TENB2 (top row) and STEAP1 (bottom row) expression as determined by immunohistocemical staining on LuCaP35V, LuCaP70, LuCaP77, and LuCaP96.1 patient-dervied xenografts. **B.** Fluorescence-activated cell sorting (FACS) analysis of TENB2 (top row) and TENB2 expression (bottom row) in LuCaP35V, LuCaP70, LuCaP77, and LuCaP96.1 tumors. Cells were isolated following the disaggregation of solid tumors grown in mice. Two tumors were studied for each type. The data for these replicates are shown in blue and green while the FACS reference standard included with each sample is shown in red.

Antigen expression results are shown in Figure 4. Representative data samples from each group are shown. Expression of TENB2 was highest in LuCaP77 tumors (3+), moderate in LuCaP70 (1-2+) and LuCaP96.1 (1+), and lowest in LuCaP35V tumors (0 to 1+) (Figure 4A). Immunohistochemistry scoring of STEAP1 was highest in LuCaP70 tumors (3+), moderate in LuCaP35V (2+) and LuCaP77 (2-3+) tumors, and lowest in LuCaP96.1 tumors (1+). Figure 4B shows different expression levels of TENB2 and STEAP1 in each cell line as determined by FACS analysis of tumor cells. Blue and green colored lines each represent an individual sample while red colored curves represents the secondary mAb standard as control. Comparable results were found for TENB2 and STEAP1 expression as determined by immunohistochemistry and FACS analysis.

An overview of the combined results of tumor growth inhibition, ^111^In-mAb tumor uptake, ^89^Zr-mAb tumor uptake and target expression as determined by immunohistochemistry and FACS is presented in Table 1 for TENB2 and in Table 2 for STEAP1.

**Table 1:** Overview of TENB2 results

Tumor	^111^In-anti-TENB2 (%ID/g)	^111^In-anti-gD (%ID/g)	^89^Zr-anti-TENB2 (%ID/g)	^89^Zr -anti-gD (%ID/g)	% Tumor growth inhibition	TENB2 expression
LuCaP35V	12.2 ± 1.6	3.9 ± 0.1	18.0 ± 1.1	5.8 ± 0.6	20	0-1+
LuCaP70	12.3 ± 0.3	3.7 ± 0.2	8.5 ± 0.9	3.8 ± 0.1	100	1-2+
LuCaP77	96.1 ± 2.8	4.1 ± 0.3	34.2 ± 1.6	8.6 ± 0.5	55	3+
LuCaP96.1	13.8 ± 0.3	3.1 ± 0.2	13.2 ± 0.2	6.2 ± 0.1	75	1+

**Table 2:** Overview of STEAP1 results

Tumor	^111^In-anti-STEAP1 (%ID/g)	^111^In-anti-gD (%ID/g)	^89^Zr-anti-STEAP1 (%ID/g)	^89^Zr -anti-gD (%ID/g)	% Tumor growth inhibition	STEAP1 expression
LuCaP35V	38.7 ± 1.5	3.9 ± 0.1	25.0 ± 0.6	5.8 ± 0.6	100	2+
LuCaP70	8.2 ± 0.3	3.7 ± 0.2	7.6 ± 0.3	3.8 ± 0.1	100	3+
LuCaP77	18.8 ± 0.6	4.1 ± 0.3	11.9 ± 0.3	8.6 ± 0.5	0	2-3+
LuCaP96.1	4.7 ± 0.3	3.1 ± 0.2	6.5 ± 0.2	6.2 ± 0.1	0	1+

## DISCUSSION

To the best of our knowledge, this is the first study to demonstrate a correlation between tumor uptake of a ^89^Zr antibody and ADC tumor growth inhibition. In four different patient-derived prostate cancer models, with varying TENB2 and STEAP1 expression, immunoPET predicts MMAE-conjugated ADC treatment efficacy.

ADC treatment is a promising new approach for anti-cancer treatment. However, without imaging to confirm good mAb uptake, disappointing response rates might be attributed to any combination of poor tissue penetration, absence of target, failure to bind target, lack of internalization, or drug resistance [1]. A lack of mAb uptake not explained by pharmacokinetic exposure may indicate a poor choice of target antigen or indicate a need to revise the mAb molecule. In contrast, a lack of drug efficacy observed in spite of visualizing strong tumor uptake may indicate the presence of active drug resistance mechanisms and suggest the need to try a different toxin.

In LuCaP35V tumors the anti-TENB2-MMAE ADC was ineffective which correlated with the low tracer uptake and target expression. In LuCaP70, LuCaP77 and LuCaP96.1 tumors anti-TENB2-MMAE ADC was efficacious, showing tumor growth inhibition. These tumors had at least moderate expression of TENB2 based on *ex vivo* immunohistochemistry and FACS tumor analysis. However, in the LuCaP77 tumor model, despite particularly high levels of ^111^In-TENB2 uptake, ADC efficacy was relatively poor as tumors started to regrow. Based on the observed tumor uptake level of 96 %ID/g (Figure 2) approximately 800 nmol of TENB2 mAb was delivered per kg of tumor tissue. This should have delivered MMAE considerably in excess of the IC50 concentration range for free toxin of 0.2 to 2 nM determined *in vitro* [3]. Even allowing for partial deconjugation of the ADC in circulation and rapid loss of MMAE catabolites from the tumor tissue, this still suggests that the amount of MMAE delivered to the tumor tissue should have been sufficient to inhibit tumor growth. Poor MMAE efficacy in the LuCaP77 model may implicate some MMAE-selective resistance mechanism mediated by certain efflux pumps or multidrug resistance of these tumors [16, 17].

STEAP1 expression also correlated with ADC treatment effect, as LuCaP35V and LuCaP70 tumors were sensitive to anti-STEAP1-MMAE ADC treatment. In the LuCaP70 model ^111^In-anti-STEAP1 uptake was the lowest (8.2 %ID/g) accompanied by tumor growth inhibition. This level of MMAE delivery was an order of magnitude less than that discussed above for anti-TENB2 in LuCap77 tumors, but was clearly sufficient to result in potency in sufficiently sensitive tumors.

While LuCaP77 tumors show high expression of TENB2 and STEAP1 these tumors did not respond to therapy. No metric of target expression predicted the degree of drug resistance that was encountered in these tumors. With the immunoPET data, there is powerful evidence of active tumor delivery of mAb *in vivo,* which provides a rationale for considering the use of the same mAb armed with alternative toxins, or entirely different cell-death effector moieties such as radioisotopes [18]. Although the presence of a receptor does not preclude resistance in clinical practice, establishing the presence or absence of an antigen is of tremendous importance. When there is no cellular uptake of ^89^Zr-mAb, no efficacy of mAb-MMAE can be expected. Clearly, the negative predictive value is greater than the positive predictive value, which is especially the case for ADCs. Therefore, establishing tumor uptake and TENB2 or STEAP1 presence may well have value in choosing appropriate treatments in the future.

In conclusion, quantitative data from immunoPET measuring relative mAb uptake patterns of TENB2- and STEAP1-targeting mAbs predict to a degree tumor growth inhibition by an ADC. ImmunoPET's capacity to demonstrate the essential aspects of ADC delivery, binding and internalization *in vivo* offers advantages complementary to existing tools. ImmunoPET may thus help confirm the necessary prerequisites for efficacy with particular mAb-target combinations. It may also identify changes in target expression or function (internalization) from genetic or treatment-induced effects. These studies were sufficiently encouraging to enter a research collaboration with Memorial Sloan Kettering Cancer Center to advance this preclinical research into phase I clinical studies of ^89^Zr-anti-STEAP1 uptake in metastatic castration resistant prostate cancer patients (www.clinicaltrials.gov, #NCT01774071) [19].

## MATERIALS AND METHODS

### Ethics statement

All applicable international, national and/or institutional guidelines for the care and use of animals were followed.

### Antibody-drug conjugates

For tumor growth inhibition studies, mAbs against TENB2 (Pr1, affinity 2.3 nM) and STEAP1 (MSTP2109A; affinity 2.4 nM) were conjugated with the auristatin moiety MC-vc-PAB-MMAE as previously described [3]. These mAbs were engineered to have exactly two site-specific thiol residues available for conjugation [20]. The average drug-to-mAb ratio was 1.8-2.0 MMAE per mAb. The HSV-1 viral coat protein gD was used as non-binding isotype control reagent (anti-gD) as this antigen is absent in mice. Anti-gD was also conjugated with MMAE. Toxin conjugation via the engineered thiols was previously shown not to significantly alter their antigen binding or pharmacokinetics [21].

### Radiolabeling of TENB2, STEAP1 and gD targeting mAbs

For *ex vivo* tumor uptake measurements, naked mAbs (without MMAE) were labeled. The naked mAb was chosen because the biology being interrogated (delivery, target binding, internalization) is independent of the drug conjugation, whereas using the ADC for radiolabeling introduces additional technical and logistical challenges to the experiment with essentially no benefit.

Indium-111 (^111^In) chelation was performed using 2-(4-isothiocyanatobenzyl)-diethylenetriaminepentaacetic acid (DTPA; Macrocyclics) by methods previously described [22]. For immunoPET imaging, naked mAbs (without MMAE) were conjugated through side-chain lysines with a desferrioxamine B (Macrocyclics) chelating group using the benzyl isothiocyanate reagent and radiolabeled with ^89^Zr [7, 23, 24].

Quality control of the conjugated and radiolabeled mAbs was performed using size exclusion high-pressure liquid chromatography, measuring radiochemical purity and the presence of aggregates or fragments. Retention of antigen binding was tested using enzyme-linked immunosorbent assays (ELISA). For this a microplate was coated with TENB2 or STEAP1 extracellular domain. After coating, 0.5 mg/ml of bovine serum albumin (BSA) in PBS was added to the microplates and incubated at 4°C for 1 h. After washing, the conjugated mAbs were added in concentrations ranging from 40 ng/ml to 0.066 ng/ml (in PBS + 0.05% BSA) and incubated for 1 h at ambient temperature. Thereafter 100 μl of 5 ng/ml anti-IgG Fab-HRP was added to each microplate well and incubated for 1 h. After washing, 100 μl of SureBlue Reserve^TM^ was added to each microplate well for 15 min at ambient temperature. Before measuring the optical density at 450 nm for detection and 650 nm for absorbance, 100 μl of 0.6 N sulfuric acid was added. A 4-parameter logistic curve fitting program was used to generate separate curves for each control (not conjugated) or conjugated mAbs. Based on this the potency was calculated.

### Patient-derived xenograft models

Patient-derived human prostate cancer tissues (LuCaP35V, LuCaP70, LuCaP77, and LuCaP96.1) were kindly provided by Dr. R. Vessella (University of Washington). LuCaP35V is an androgen independent variant of LuCaP35 isolated from an inguinal lymph node metastasis [25]. LuCaP70, LuCaP77 and LuCaP96.1 were derived from liver, femur metastases or prostate tissue, respectively [26]. The patient-derived tumors have varying expression of TENB2 and STEAP1 that was determined *ex vivo,* as described below.

The LuCaP70, LuCaP77 and LuCaP96.1 patient derived xenografts were established through serial subcutaneous implantations of 20-30 mm^3^ tumor fragments in intact male CB17 SCID-beige mice. LuCaP35V was propagated in castrated mice (Charles River Laboratories). For all experiments mice were age matched and inoculated with the same tumors and designated into separate cohorts. Experiments were conducted more than a year apart in time and consequently on cohorts of tumors with somewhat different numbers of passages and growth properties because of the logistics of working with patient-derived xenograft models.

All animal studies were conducted in accordance to the guidelines of the Institutional Animal Care and Use Committee at Genentech, Inc.

### Dose-escalation pilot studies of ^111^In-labeled anti-STEAP1

The dose escalation study of ^111^In-anti-TENB2 was previously reported [23]. For the dose escalation study of ^111^In-anti-STEAP1, LuCAP35V tumor bearing mice were dosed intravenously with approximately 0.18 MBq ^111^In-labeled anti-STEAP1 (90 MBq/mg). Labeled tracer was mixed with cold mAb protein to achieve final dose levels of 0.08 mg/kg, 0.2 mg/kg, 1 mg/kg, 10 mg/kg, or 40 mg/kg of STEAP1 per mouse (n = 5 per group). Tissues were harvested three days post-injection for gamma counting with the Wallac Wizard 1480 Automatic Gamma Counter (Perkin-Elmer).

### Tumor tracer uptake and ADC growth inhibition studies

Cut-and-count tumor tracer uptake studies were performed in LuCaP35V, LuCaP70, LuCaP77, and LuCaP96.1 patient-derived xenografts (n = 4-5 per group) following the intravenous injection of a single dose (5 mg/kg) of mAb targeting TENB2, STEAP1, or gD (isotype control) radiolabeled with ^111^In (0.18-0.74 MBq). *Ex vivo* analysis was performed three days post-injection of tracer to allow for tissue uptake, mAb internalization, and sufficient background clearance. Tumors were harvested, blot-dried, and weighed for gamma counting with background subtraction and decay correction against dosing solution standards.

Tumor growth inhibition was also studied in LuCaP35V, LuCaP70, LuCaP77, and LuCaP96.1 patient-derived xenografts following a single dose of 5 mg/kg (same dose as for tracer above) of the ADCs targeting TENB2, STEAP1 or gD (isotype control). This dose was chosen because it was efficacious in sensitive models yet well-tolerated by the animals in previous studies (data not shown). ADCs were administered intravenously via the tail vein (n = 8-10 per group). Tumor volume growth was monitored for up to 70 days. Tumor length (l, the longest dimension) and width (w, perpendicular to the length) were measured by calipers; tumor volume V was approximated as V = lw^2^/2.

### PET imaging

^89^Zr-PET imaging studies of tumor tracer uptake were performed after the intravenous injection of a single dose of 5 mg/kg of the mAbs targeting TENB2, STEAP1, or gD (isotype control) radiolabeled with ^89^Zr (3.7 MBq; 30 MBq/mg), in LuCaP35V, LuCaP70, LuCaP77, and LuCaP96.1 patient-derived xenografts (n = 3-5 per group). PET imaging was conducted five days later using Siemens Inveon PET/CT scanners (Siemens). Animals were lightly anesthetized for restraint with approximately 3.5% sevoflurane, and body temperature was maintained at 37°C by warm airflow. PET scans were 15-30 minute static scans.

Region of interest measurements defined by using software tools were made on multiple axial slices of the tissues using IRW software (Siemens). Decay-corrected signal intensity of tumor was measured as percentage of the injected dose per gram (%ID/g), assuming a tissue density of 1 ml per 1 gram soft tissue.

### *Ex vivo* tumor analysis

Tumor samples used for *ex vivo* characterization of target expression levels were derived from tumor fragments with the same number of passages and in the same cohort of mice and as were used for efficacy studies.

Immunohistochemical determination of antigen expression was performed on 4 μm sections from formalin-fixed paraffin-embedded tissue. The primary mAbs used for immunohistochemistry were chosen because of their ability to detect antigen in fixed tissue and they were not the same mAbs used in experiments with live animals or unfixed tissues. Staining for TENB2 was performed on the Discovery XT autostainer (Ventana Medical Systems). Tissue sections were incubated in 2.5 μg/ml humanized anti-TENB2 primary mAb, clone PR-1, and then in 7.5 μg/ml goat anti-human, biotin conjugated mAb (Vector Laboratories). Human IgG (Jackson Immunoresearch) was used as a negative control mAb. Detection was performed utilizing Ventana DabMAP. For STEAP1, tissue sections were incubated in 10 μg/ml sheep primary mAb and then in biotinylated donkey anti-sheep IgG (Jackson Immunoresearch). Tissue sections were treated with Ventana DABMap™ for the detection of signals and visualization through 3,3′-diaminobenzidine as a chromogenic peroxidase substrate. Immunohistochemistry scoring was performed by a board-certified pathologist and is based on the intensity of positive staining observed in at least 50% of tumor cells: “negative” indicates no detectable signal, “1+” indicates a weak signal, “2+” indicates a moderate signal, and “3+” indicates a strong signal.

For fluorescence-activated cell sorting (FACS) experiments, tumor tissues were harvested, chopped and macerated in cell dissociation buffer (Gibco-BRL, #13151014), strained and washed with FACS buffer (1% FBS and 2mM EDTA in PBS). Cells were centrifuged at 4°C and resuspended in 20 ml ACK lysis buffer (163 mM NH_4_Cl, 10 mM KHCO_3_, 0.1 mM EDTA pH 7.4) again centrifuged and resuspended into FACS buffer before being dispensed into a 96-well plate where they were incubated for one hour on ice with 3 mg/ml mAbs (unlabeled mAbs targeting TENB2 or STEAP1). After washing, 2 mg/ml of a secondary anti-human IgG-Fc domain PE mAb was added and incubated on ice for an additional hour. Cells were washed three times and resuspended in 100 ml of FACS buffer with 0.5 mg/ml PI and analyzed using a FACS Calibur Flow Cytometer (BD).
